# Krüpple-like factors in cardiomyopathy: emerging player and therapeutic opportunities

**DOI:** 10.3389/fcvm.2024.1342173

**Published:** 2024-03-07

**Authors:** Le-Kun Gui, Huang-Jun Liu, Li-Jun Jin, Xiao-Chun Peng

**Affiliations:** ^1^ Department of Cardiology, The First Affiliated Hospital of Yangtze University, Jingzhou, Hubei, China; ^2^ School of Medicine, Yangtze University, Jingzhou, Hubei, China; ^3^ Department of Pathophysiology, School of Basic Medicine, Health Science Center, Yangtze University, Jingzhou, Hubei, China; ^4^ Laboratory of Oncology, School of Basic Medicine, Center for Molecular Medicine, Health Science Center, Yangtze University, Jingzhou, Hubei, China

**Keywords:** Krüppel-Like Factors, cardiomyopathy, genetic screen, diabetic cardiomyopathy, heart failure

## Abstract

Cardiomyopathy, a heterogeneous pathological condition characterized by changes in cardiac structure or function, represents a significant risk factor for the prevalence and mortality of cardiovascular disease (CVD). Research conducted over the years has led to the modification of definition and classification of cardiomyopathy. Herein, we reviewed seven of the most common types of cardiomyopathies, including Arrhythmogenic Right Ventricular Cardiomyopathy (ARVC), diabetic cardiomyopathy, Dilated Cardiomyopathy (DCM), desmin-associated cardiomyopathy, Hypertrophic Cardiomyopathy (HCM), Ischemic Cardiomyopathy (ICM), and obesity cardiomyopathy, focusing on their definitions, epidemiology, and influencing factors. Cardiomyopathies manifest in various ways ranging from microscopic alterations in cardiomyocytes, to tissue hypoperfusion, cardiac failure, and arrhythmias caused by electrical conduction abnormalities. As pleiotropic Transcription Factors (TFs), the Krüppel-Like Factors (KLFs), a family of zinc finger proteins, are involved in regulating the setting and development of cardiomyopathies, and play critical roles in associated biological processes, including Oxidative Stress (OS), inflammatory reactions, myocardial hypertrophy and fibrosis, and cellular autophagy and apoptosis, particularly in diabetic cardiomyopathy. However, research into KLFs in cardiomyopathy is still in its early stages, and the pathophysiologic mechanisms of some KLF members in various types of cardiomyopathies remain unclear. This article reviews the roles and recent research advances in KLFs, specifically those targeting and regulating several cardiomyopathy-associated processes.

## 1 Introduction

Cardiomyopathies are a group of heterogeneous pathological disorders characterized by alterations in cardiac structure and function (1). Their conception can be traced to Fiedler's discovery of a series of fatal cases of cardiac hypertrophy and Heart Failure (HF) in young people in 1899 (2). With advancements in medicine and an enhanced understanding of diseases, cardiomyopathies have been updated and categorized into several groups (1, 3–5), including Dilated Cardiomyopathies (DCM), Restrictive Cardiomyopathies (RCM), Hypertrophic Cardiomyopathies (HCM) and Arrhythmogenic Right Ventricular Cardiomyopathies (ARVC), among others (3, 6). Furthermore, research is expanding into the disease(cardiomyopathy)-causing genes (7, 8). Cardiomyopathy etiology is multifactorial and has not been fully elucidated hitherto. The molecular mechanisms underlying cardiomyopathy-associated myocardial remodeling and cardiac dysfunction are highly complex and warrant further research. In recent years, the Krüppel-Like Transcription Factors (KLFs) family has gained renewed attention as research advances have revealed the involvement of KLFs in various processes, including cardiomyopathy progression. Krüppel-Like Factors (KLFs) are a group of DNA-binding proteins first discovered in the early 1990s as erythroid cell-specific Transcription Factors (TFs) (9). As important gene transcription regulators, KLFs are involved in multiple processes regulating the occurrence and development of myocardial diseases, including Oxidative Stress (OS), inflammatory responses, and myocardial hypertrophic fibrosis, as well as cell proliferation, differentiation, apoptosis, and regeneration (10–14), via amino acid terminal region regulation of protein-DNA and Protein-Protein Interactions (PPIs). Although research has revealed key insights on KLFs and their involvement in cardiomyopathies, much is unknown about the KLF-induced pathophysiologic alterations in cardiomyopathies and the potential therapeutic targets for treating these diseases. Therefore, this article aims to summarize the current roles and molecular mechanisms of KLF family members in different types of cardiomyopathies and to outline the key roles KLFs play in Cardiovascular Diseases (CVDs).

## 2 Krüppel-Like Factors

**Figure 1:**
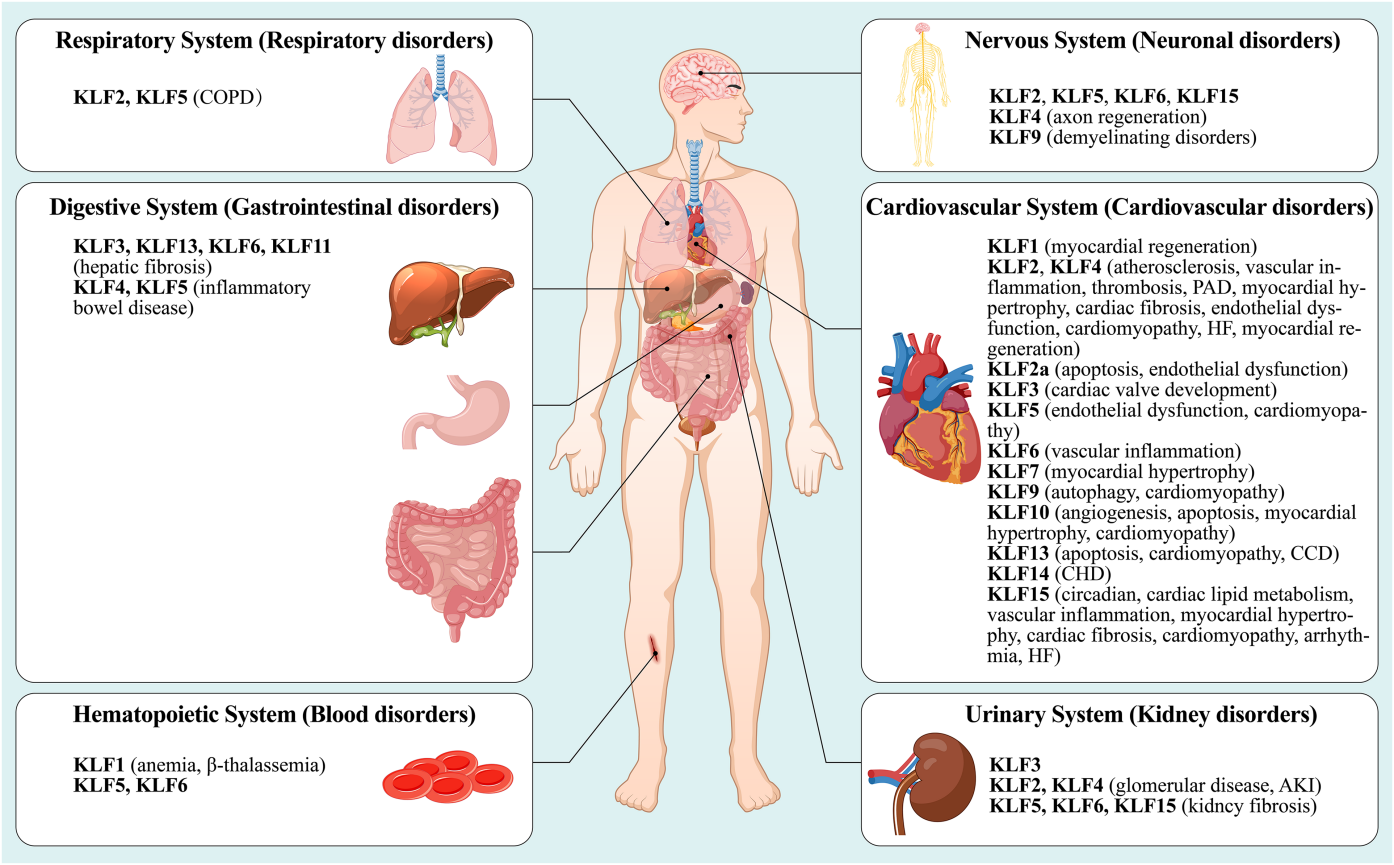
A summary nof the type distribution of KLFs in humans and their roles in diseases. The figure describes the physiological distribution of KLFs in various body systems (cardiovascular system, respiratory system, nervous system, urinary system, digestive system and hematopoietic system), and its role in the initiation and development of the corresponding pathologic diseases or disorders. See text for details. PAD, peripheral arterial disease; HF, heart failure; CCD, congenital cardiovascular diseases; CHD, coronary heart disease; COPD, chronic obstructive pulmonary disease; AKI, acute kidney injury.

Although KLFs are found in multiple organ systems, including the cardiovascular, respiratory, gastrointestinal, urinary, neurological, and hematopoietic systems (15), their tissue expression varies (Figure 1). In other words, some family members are universally expressed, while others are specifically expressed (16–18). Studies have shown that KLFs possess three highly conserved C2H2 zinc-finger domains in their carboxy-terminal region. These domains facilitate interactions with common GC-rich sites during transcriptional regulation, enabling them to activate or inhibit cellular development (17, 19). It has been reported that KLF proteins show homology in their gene sequences, with the structural similarity allowing for overlapping transcriptional targets. For example, KLF2 is expressed in the cardiovascular, respiratory, urinary, and nervous systems, while KLF5 is found in the cardiovascular, gastrointestinal, urinary, nervous, and hematopoietic systems. On the other hand, KLF6 can be found in all of the above-mentioned systems. However, KLF proteins possess unique amino-terminal sequences that provide specific regions for interaction with distinct binding partners. For instance, KLF1 features a minimal transactivation domain (TAD) within its first 100 amino acids. Research has classified KLF1 TAD into two functional subdomains, TAD1 and TAD2, with the latter conserved in four additional KLF proteins (KLF2, 4, 5, and 15) (20). KLF1 is predominantly expressed in mast and erythroid cells and is associated with β-thalassemia (21), whereas KLF2 is highly expressed in the lungs and is an essential regulator involved in lung development (22, 23). On the other hand, KLF15 is abundantly expressed in the cardiovascular system and is a negative regulator of cardiac hypertrophy, ensuring appropriate cardiac responses to physiological stress signals (24). Additionally, KLF3, 8, and 12 are characterized by an N-terminal repression domain containing a CtBP recognition motif (25–27), while the N-terminal regions of KLF9, 10, 11, 13, 14, and 16 contain a Cabut domain with a Sin3 interaction domain (SID), serving as a transcriptional regulatory repression domain (28–32).

Krüppel-Like Factors (KLFs) represent a conserved family of TFs, with the initial discovery of KLF1 (EKLF) in erythrocytes in 1993 (9). Since then, researchers have identified 18 members (KLF1-KLF18) in mammals. However, some debate surrounds whether there are only 17 KLF members, excluding KLF18, which is considered a duplicate of KLF17 (33). These KLF proteins can be classified into three groups based on their functional characteristics (Table 1). Group 1 includes KLFs 3, 8, and 12, acting as transcriptional repressors that interact with CtBP (C-terminal binding protein) (17, 26, 34, 35). Group 2 consists of KLFs 1, 2, 4, 5, 6, and 7, primarily functioning as transcriptional activators (9, 36–40). In contrast, KLFs 15, 17, and 18 form the non-consensus group, exhibiting more distant relationships and lacking clear protein interaction motifs (17, 24, 33, 46). The remaining KLFs, categorized as group 3 members, exert repressive effects similar to those in group 1 but their effects are dependent on its interaction with the transcriptional co-repressor Sin3A (15, 41–45).

**Table 1:** Phylogenetic classification of Krüppel-Like Factors.

Group members		Alternative names	Chromosome localization	Characteristics	Function	References
Group 1	KLF3	BKLF	4p14	Presence of CtBP-binding sites	The C-terminal domain binds the CtBP protein to mediate transcriptional repression.	(17, 26, 34, 35)
	KLF8	BKLF3/ZNF741	Xp11.21			
	KLF12	AP-2rep/AP2REP/HSPC122	13q22.1			
Group 2	KLF1	EKLF	19p13.13	Ability to bind deacetylases	Convenes acetyltransferase activity factors (CBP, p300, and P/CAF) to function as transcriptional activators while promoting chromatin remodeling.	(9, 10, 36–40)
	KLF2	LKLF	19p13.11			
	KLF4	GKLF/EZF	9q31.2			
	KLF5	IKLF/BTEB2/CKLF	13q22.1			
	KLF6	BCD1/CBA1/ CPBP/COPEB/GBF/PAC1/ST12/ZF9	10p15.2			
	KLF7	UKLF	2q33.3			
Group 3	KLF9	BTEB/BTEB1	9q21.12	Presence of a Sin3A-binding sites	Interacts with the transcriptional co-repressor Sin3A to achieve inhibitory activity.	(15, 41–45)
	KLF10	TIEG/TIEG1/EGRα/EGRA	8q22.3			
	KLF11	TIEG2/Tieg3/FKLF/FKLF1/MODY7	2p25.1			
	KLF13	FKLF2/BTEB3/RFLAT-1/RFLAT1/NSLP1	15q13.3			
	KLF14	BTEB5	7q32.2			
	KLF16	DRRF/BTEB4/NSLP2	19p13.3			
No consensus group	KLF15	KKLF	3q21.3	Distantly related and contain no defined protein interaction motifs	Interaction domains remain undetermined.	(17, 24, 33, 46)
	KLF17	ZLF393/ZNF393/ZFP393	1p34.1			
	KLF18	KLF18	1p34.1			

## 3 Cardiomyopathy

Cardiomyopathy manifests in various ways, ranging from microscopic changes in myocardial cells, to fulminant HF with inadequate tissue perfusion and arrhythmias caused by electrical conduction abnormalities (47). Cardiomyopathies, which means myocardium diseases, were traditionally categorized as hypertrophic, dilated, and restrictive. However, advances in genomics have demonstrated the diversity in their phenotypic expression (1).

**Figure 2:**
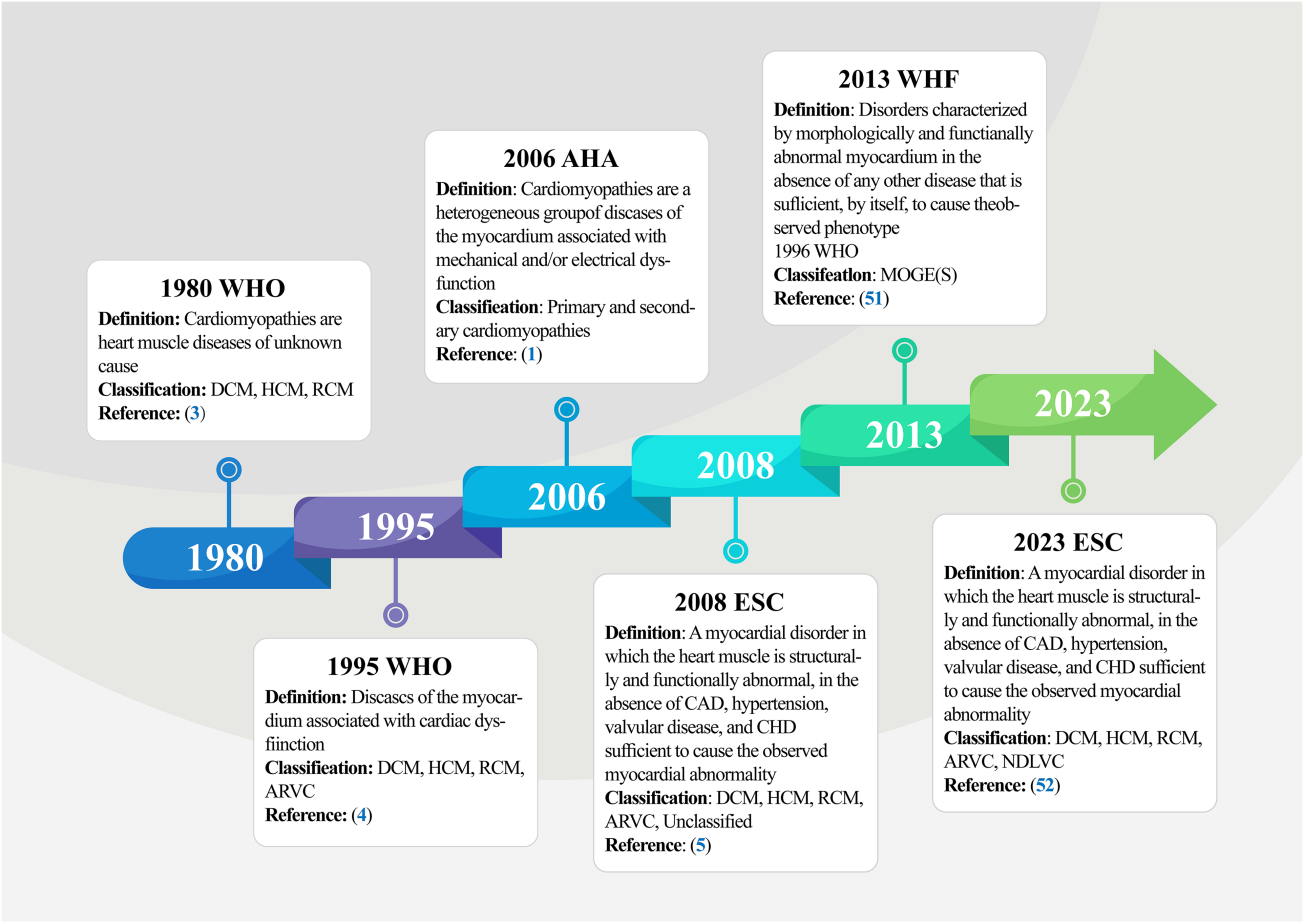
Timeline of definitions and classifications of cardiomyopathy. The figure summarizes the five main stages involved in the initiation and progression of cardiomyopathy, from the formal naming of cardiomyopathies and primary classification by the WHO in 1980 to the current more comprehensive definition and classification of cardiomyopathy by the ESC in 2023. WHO, world health organization; DCM, dilated cardiomyopathy; HCM, hypertrophic cardiomyopathy; RCM, restrictive cardiomyopathy; AVRC, arrhythmogenic right ventricular cardiomyopathy; AHA, American heart association; WHF, world heart federation; ESC, European society of cardiology; CAD, coronary artery disease; CHD, congenital heart disease.

In 1980, the World Health Organization (WHO) published its first report on cardiomyopathies (3), defining them as muscle diseases of unknown cause and categorizing them as DCM, RCM, and HCM. This diagnostic criterion was updated by subsequent classification iterations, leading to the WHO amending the definition of cardiomyopathy in 1995 (4), redefining it as myocardial illnesses associated with cardiac dysfunction, including ARVC in the previous classification, and further elucidating and highlighting the term “specific cardiomyopathy”. Based on previously published clinical practice guidelines and recent advances in the characterization of myocardial diseases (48–50), the American Heart Association (AHA) Committee proposed a new, more rigorous classification in 2006 that reflects the evolving molecular genetics of cardiology and standardizes nomenclature inconsistencies (1). The panel proposed a new definition of cardiomyopathies, describing them as a heterogeneous group of myocardial diseases with etiologies related to mechanical and electrical dysfunction, often exhibiting inappropriate ventricular hypertrophy and/or dilatation and arising from multiple causes, often genetic. Regarding classification, the expert consensus panel recommended the categorization of cardiomyopathies as primary or secondary. Primary cardiomyopathies referred to diseases occurring exclusively or predominantly in the myocardium, while secondary cardiomyopathies described pathologic myocardial illnesses associated with a multisystem disease. Primary cardiomyopathies were further grouped into the genetic, mixed, and acquired classes, with genetic cardiomyopathies including HCM and ARVC; mixed cardiomyopathies including genetic and non-genetic DCM; and acquired cardiomyopathies (also known as inflammatory cardiomyopathies) including myocarditis. Notably, ion channelopathies were also listed as primary cardiomyopathies in the scientific statement. Adding to the ongoing updates, the European Society of Cardiology (ESC) introduced a new cardiomyopathy classification criteria in 2008 (5). This classification criteria were oriented towards clinical utility, was based on ventricular structure and function, and defined cardiomyopathies as structural and/or functional abnormalities of the myocardium not caused by Coronary Artery Diseases (CADs), Hypertension (HTN), valvular diseases, and congenital heart defects. Based on morphologic and functional characteristics, cardiomyopathies were categorized into five groups (DCM, RCM, HCM, ARVC, and unclassified), each of which was further divided into several subtypes, including familial (genetic), non-familial (non-genetic), and unidentified gene defect classes, as well as disease sub-types, and idiopathic subgroups. Notably, this version does not distinguish between primary and secondary cardiomyopathies. In 2013, Arbustini and other cardiovascular experts proposed a novel set of phenotypic-genotypic MOGE(S) classification criteria for cardiomyopathies (51), which was supported by the World Heart Federation (WHF). This classification criteria described cardiomyopathies as diseases characterized by morphologically and/or functionally abnormal myocardium, devoid of disruptions caused by the clinical manifestations of other diseases. The criteria classify cardiomyopathic disorders based on five characteristics: M (Morphofunctional features), O (Organ involvement), G (Genetic or familial inheritance patterns), E (Clear etiologic annotations), and optionally, S (Functional status information). The most current version is the *2023 ESC Guidelines for the Management of Cardiomyopathies* (52). Here, the ESC defines cardiomyopathy as a disease of the myocardium with structural and functional abnormalities. It should be noted that the presence of such a disease does not preclude the occurrence of other diseases, such as CADs, HTN, and valvular and congenital heart diseases. The guideline task force updated the description of the phenotype of non-dilated left ventricular cardiomyopathy (NDLVC) and did not recommend both left ventricular non-compaction (LVNC) as well as Takotsubo syndrome (stress cardiomyopathy) as separate subtypes and no longer uses arrhythmogenic cardiomyopathy (ACM, from the original terminology of ARVC) as a distinct cardiomyopathic subtype. The guidelines further highlight the diagnostic value of multimodality imaging and the prognostic importance of genetic testing, recommend a multidisciplinary team approach to the management of cardiomyopathy that focuses on the patient and his or her family, and recommend clinical evaluation and genetic cascade screening for relatives of patients with cardiomyopathy (Figure 2). Although there are various types of cardiomyopathies, our understanding of these diseases and their current typing methods remains limited. However, with progress in cardiomyopathy research, future cardiomyopathy definitions and typing methods will be more refined and clinically applicable.

## 4 KLFs and cardiomyopathy

**Figure 3:**
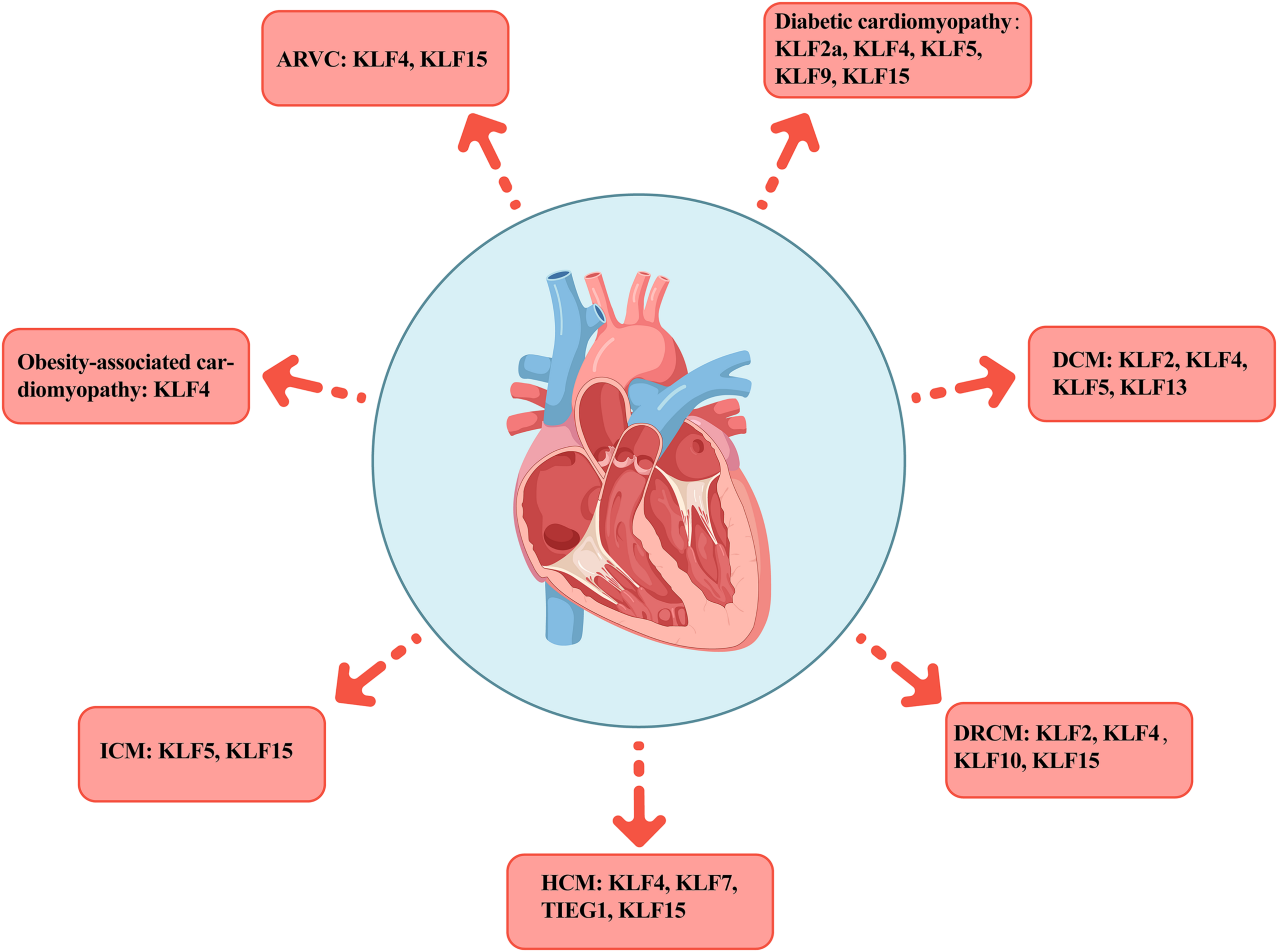
Distribution of KLFs across various cardiomyopathies. The figure presents seven typical cardiomyopathies including AVRC, diabetic cardiomyopathy, DCM, DRCM, HCM, ICM, and obesity cardiomyopathy, as well as the distribution of the KLFs family members in each subtype. AVRC, arrhythmogenic right ventricular cardiomyopathy; DCM, dilated cardiomyopathy; DRCM, desmin-related cardiomyopathy; HCM, hypertrophic cardiomyopathy; ICM, ischemic cardiomyopathy.

Many KLFs are involved in cardiovascular system regulation and in diversely controlling cell, tissue, and system metabolism. For example, KLFs 2 and 4 act as nodal regulators of endothelial function, promoting anti-inflammatory and anti-thrombotic gene expression, which collectively keep blood vessels healthy (53, 54). However, in pathological states, the heart undergoes cardiac remodeling due to disease and stress-induced long-term metabolic changes, triggering structural and functional abnormalities of the myocardium that eventually lead to cardiomyopathy. Cardiomyopathies are a heterogeneous group of pathological conditions. As some of the TFs affecting various pathophysiological processes in myocardial diseases, KLFs play vital roles in different cardiomyopathies (Table 2, Figure 3).

**Table 2:** Role of KLFs family members in various cardiomyopathies.

Cardiomyopathy	KLF Involved	Effect	Mechanism	References
Arrhythmogenic right ventricular cardiomyopathy	KLF4	Inhibit	Mitogen-activated kinase kinase-7 deficiency leads to separation of KLF4 from promoter regions of potassium channel genes, resulting in reduced transcription levels and delayed repolarization.	(55–57)
	KLF15	Protector	KLF15 transcriptionally controls myocardial energy metabolism and rhythmic expression of Kv channel interacting protein 2.	(58–60)
Diabetic cardiomyopathy	KLF2a	Protector	Reducing phosphorylated AMPK increases p53 expression, leading to the decrease in KLF2a, which promotes CMs apoptosis and induces cardiac remodeling and/or cardiac dysfunction.	(61)
	KLF4	Promoter	AEG-1 aggravated autophagy through upregulating KLF4.	(62)
	KLF5	Promoter	FOXO1 increases the expression of KLF5, which causes oxidative stress and contributes to diabetic cardiomyopathy by inducing NADPH oxidase (NOX)4 promoter expression and ceramide accumulation.	(11)
		Protector	Inhibition of KLF5, the positive transcriptional regulator of cardiac Ppara, leads to cardiac dysfunction, and cardiomyocyte-specific ablation of KLF5 decreases cardiac ATP and FAO levels, leading to cardiac insufficiency.	(63–67)
	KLF9	Promoter	Upregulation of KLF9 worsens cardiac function, exacerbating oxidative stress, inflammatory responses, and hypertrophic myocardial fibrosis.	(68, 69)
		Promoter	The miR-30d/KLF9/VEGFA pathway and the KLF9/VEGFA pathway can regulate the autophagy level in diabetic rats.	(70)
	KLF15	Reduction	KLF15 negatively regulates cardiac fibrosis through SDF-1β in type 2 diabetes.	(24, 71–73)
Dilated cardiomyopathy	KLF2	Reduction	Targeting CCR2 protein inhibits bone marrow mobilization of Ly6C^high^ monocytes and extraction of EVs from KLF2 gene-overexpressing ECs reduces cardiac inflammatory response and ameliorates left ventricular dysfunction.	(74)
	KLF4	Inhibit	Transfection with Sendai virus carrying KLF4, OCT3/4, Sox2, and c-Myc genes reprogrammed to generate iPSCs.	(56, 75)
	KLF5	Mutations	Mutations disrupt the synergistic transactivation between KLF5 and NF-κB1, predisposing mutation carriers to DCM.	(76)
	KLF13	Mutations	Three mutations in the KLF13 gene cosegregate with the DCM phenotype and are complete penetrance.	(77)
Desmin-related cardiomyopathy	KLF2	Protector	Extracellular signal-regulated kinase 5 signaling pathway can induce the upregulation of KLF2 gene via the Sp1 transcription factor.	(78)
	KLF4	Inhibit	Reprogramming factors KLF4, OCT4, SOX2, CMYC were delivered using Sendai viruses.	(79)
	KLF10	Promoter	KLF10 inhibits myoblast proliferation by suppressing the function of pro-proliferative signaling molecules and the expression of cyclin.	(80, 81)
	KLF15	Protector	KLF15 can stimulate the expression of the slow-twitch fiber gene Myh7 by targeting the nuclear factor of activated T-cells and cytoplasmic 1 gene.	(82)
Hypertrophic cardiomyopathy	KLF4	Inhibit	KLF4 negatively regulates cardiac hypertrophy as a transcriptional control center for cardiac metabolic function and mitochondrial life cycle.	(83–85)
	KLF7	Protector	KLF7 regulates enzymes in glycolysis and fatty acid oxidation to attenuate metabolic imbalances caused by cardiac hypertrophy.	(86)
	TIEG1 (3 zinc ﬁnger family of KLF10)	Promoter	TIEG1 mediates TGFb by regulating the Smad signaling pathway to achieve cell proliferation inhibition and induce apoptosis.	(87, 88)
	KLF15	Protector	Single nucleotide polymorphisms in KLF15 are strongly related to cardiac hypertrophy, and their deletion or inhibition leads to left ventricular hypertrophy in diabetic patients.	(58, 89, 90)
Ischemic cardiomyopathy	KLF5	Promoter	KLF5 induces SPTLC1 and SPTLC2 expression and increases myocardial ceramide levels, ventricular dysfunction and eccentric remodeling, and exacerbates ischemic heart failure.	(91)
	KLF15	Reduction	Upstream regulator KLF15 regulated by EZH2 in a SET domain-dependent manner.	(92)
		Protector	KLF15 inhibits p53 function by lowing abundance of acetylated p53 during KLF15-p53-p300 pathway.	(93–95)
		Reduction	KLF15 regulates increased transcription of genes involved in cardiac remodeling, and KLF15 expression is significantly reduced in ischemic hearts.	(72, 92, 96)
Obesity-associated cardiomyopathy	KLF4	Protector	KLF4 contributed to berberineinduced cardiac mitochondrial benefits and lipid metabolism.	(97–99)

### 4.1 Arrhythmogenic right ventricular cardiomyopathy

It has been reported that Arrhythmogenic right ventricular cardiomyopathy (ARVC) is an autosomal dominant cardiomyopathy (100) characterized by cardiomyocyte replacement with fibro-adipose tissues, resulting in abnormal Excitation-Contraction (EC) coupling and a series of malignant events such as Ventricular Arrhythmias (VA), HF, and Sudden Cardiac Death (SCD) (101, 102). The disease is considered a major cause of sudden death in young adults, especially athletes (6, 103), and its prevalence is estimated to be between 1/2,500 and 1/5,000, with a male predominance (female to male ratio of 1:2.7), which may be related to the disease genes and androgens (104, 105).

Intercalated disks (connective structures between cardiomyocytes) are the functional unit that provides the mechanical and/or electrical coupling that enables the coordinated and synchronized contraction of cardiomyocytes. According to research, variants affecting the gene encoding the desmosomal protein are responsible for approximately 50%–60% of ARVC cases (106). Five of the eight genes that account for pathogenic or potentially pathogenic variants are desmosomal genes (107). The primary function of “glue” desmosomes is transmitting mechanical strength between myocardial cells. When the desmosomes are mutated, the myocardium undergoes cell detachment and death, later replaced by fibro-adipose tissues, thereby resulting in scarring, wall thinning, and aneurysms. This adhesion defect is exacerbated by exercise, with a greater impact on the thinner right ventricle (55, 108). Li et al. (58) identified a novel heterozygous mutation in the KLF15 gene in a family with atrial fibrillation (AF), ventricular arrhythmia, and hypertrophic cardiomyopathy. Subsequent investigations revealed that the loss-of-function mutation in KLF15 could potentially trigger AF by disrupting myocardial energy metabolism (59). Moreover, this mutation could exacerbate AF by prolonging repolarization and extending the effective refractory period (60, 109).

Plakophilin-2 (PKP2) mutations are the most widespread genetic association in ARVC. Khudiakov et al. (56) obtained an iPSC line carrying 2 mutations in the PKP2 gene from a 14-year-old female with severe ARVC, and detected high OCT4, NANOG, and SOX2 mRNA levels after reprogramming and transducing them with Sendai virus vectors, confirming the pluripotency of the iPSC line. Yang et al. (75) used a similar approach to transduce PBMCs carrying three reprogramming factors (KOS, KLF4, and cMYC) obtained from a 41-year-old female ARVC patient. The iPSCs obtained exhibited pluripotency marker expression, intact karyotype, and the potential to differentiate into multiple germ layers. Similarly, KLF4 produces iPSCs via reprogramming in DCM (110, 111). Drawing on KLF4's pivotal role in regulating cardiac potassium (K^+^) channels, Chowdhury et al. (57) established a connection between mitogen-activated kinase kinase-7 (MKK7) deficiency and heightened susceptibility to arrhythmia. MKK7 deficiency prevents the phosphorylation of histone deacetylase-2, leading to the accumulation of filamentin A in the nucleus. This filamentin A then forms a complex with KLF4, causing KLF4 to dissociate from the promoter regions of several potassium channel genes. Consequently, this disrupts transcription levels, delays repolarization, and ultimately precipitates ventricular arrhythmia.

### 4.2 Diabetic cardiomyopathy

Diabetic Cardiovascular Diseases (DCVDs) account for over 50% of diabetes-related mortalities (112), including diabetic cardiomyopathy (a specific type of heart disease) cases (113, 114). The existence of a specific diabetic heart muscle disease that does not involve CAD or HTN was first proposed by Lundbaek in 1954 (115, 116). In 2008, the European Society of Cardiology (ESC) defined the disease as an abnormality of the heart muscle in terms of its structure and function, along with the absence of CADs, HTN, and valvular and congenital heart diseases sufficient to cause the observed myocardial abnormality (5). Nevertheless, the ESC stated again in 2018 that there was no definition of diabetic cardiomyopathy (117). Given the lack of consensus on its definition, it remains difficult to accurately assess epidemiologic data on diabetic cardiomyopathy-related morbidity and mortality. However, clinical trials in Type 2 Diabetes Mellitus (T2DM) patients revealed an HF prevalence of 10%–30% (117).

Diabetic cardiomyopathy is a major risk factor for diabetes-related morbidity and mortality. It is characterized by hypoinsulinemia and hyperglycemia in Type 1 Diabetes Mellitus (T1DM) patients and hyperinsulinemia or insulin resistance in T2DM patients (118). Although the underlying mechanism of some KLFs in diabetes remains unclear (44), studies have acknowledged the essential role KLFs play in many types of diabetes (71, 89, 119–124) (Table 3). For example, it was reported that KLF7 can regulate insulin sensitivity and susceptibility to type 2 diabetes by lowering adiponectin and leptin levels (126, 127), and can negatively regulate miR-132-3p to aggravate the transition of Human Umbilical Vein Endothelial Cells (HUVECs) to a mesenchymal state after high glucose exposure (128). Similarly, KLF14 has been implicated in increasing susceptibility to type 2 diabetes by regulating key genes associated with insulin resistance (122, 123). For instance, the risk alleles G of rs972283 and rs4731702 have been linked to this effect. Additionally, KLF11 plays a negative regulatory role in NDM, Maturity Onset Diabetes in Young (MODY) (129, 132), as well as T2DM (130) and Type 1B Diabetes (131). Mutations in KLF11 may hinder insulin secretion in pancreatic *β*-cells by inhibiting insulin promoter regulatory activity, consequently impairing insulin gene transcription in NDM and MODY. Moreover, KLF11 is involved in regulating hepatic glucose metabolism. Zhang et al. (130) found that overexpression of KLF11 in mouse hepatocytes inhibited the expression of gluconeogenic genes, such as peroxisome proliferator-activated receptor γ coactivator-1α (PGC-1α) and phosphoenolpyruvate carboxykinase (PEPCK-C), thereby reducing cellular glucose output. As a negative regulator of adipogenesis, KLF2 is highly expressed in preadipocytes and regulates glucolipid metabolism and insulin sensitivity by directly inhibiting the PPARγ2 promoter activity (119). Furthermore, although KLFs 2, 4, and 9 have been strongly associated with Gestational Diabetes Mellitus (GDM) development, the specific regulatory roles of KLFs 2 and 4 remain unclear, necessitating additional research in a larger patient population (121, 125). Research indicates that KLF9 plays a crucial role in regulating hepatic glucose metabolism. Hepatic KLF9 overexpression induced by dexamethasone (Dex) and fasting directly binds to its promoter, stimulating the expression of the PGC-1α gene and activating the gluconeogenesis program. A mutation in KLF9 eliminates the stimulatory effect of Dex on cellular glucose output and effectively attenuates Dex-induced hyperglycemia (120), a critical finding for patients requiring long-term glucocorticoid therapy.

**Table 3:** Effect and mechanism of KLFs in diabetes mellitus.

Member of KLFs	Type of diabetes	Title	Authors	Years	Mechanism	Effect	References
KLF2	T2DM	The Krüppel-like factor KLF2 inhibits peroxisome proliferator-activated receptor-gamma expression and adipogenesis	Banerjee SS et al.	2003	Related to the regulation of PPARγ	Regulates glycolipid metabolism and insulin sensitivity	(119)
	GDM	Differentiated serum levels of Krüppel-Like Factors 2 and 4, sP-selectin, and sE-selectin in patients with gestational diabetes mellitus	Zhang HM et al.	2022	Low levels of KLF2 are the risk factor for GDM	Serum KLF2 may be an indicator of GDM, but its exact mechanism of action remains unknown	(125)
KLF4	GDM	Differentiated serum levels of Krüppel-Like Factors 2 and 4, sP-selectin, and sE-selectin in patients with gestational diabetes mellitus	Zhang HM et al.	2022	The serum levels of KLF4 were not significantly altered in GDM patients	Whether KLF4 plays an important regulatory role in GDM is still being explored	(125)
KLF7	T2DM	Egr1 mediates the efect of insulin on leptin transcription in adipocytes	Mohtar O et al.	2019	Reduces adiponectin and leptin levels	Regulates insulin sensitivity and is related to T2DM susceptibility	(126)
		Single nucleotide polymorphisms in the gene encoding Krüppel-like factor 7 are associated with type 2 diabetes	Kanazawa A et al.	2005	Reduces adiponectin and leptin levels	Regulates insulin sensitivity and is associated with T2DM susceptibility	(127)
		MiR-132-3p and KLF7 as novel regulators of aortic stifening-associated EndMT in type 2 diabetes mellitus	Hulshoff MS et al.	2023	MiR-132-3p is downregulated in diabetic or high glucose conditions and activates the expression of the KLF7	KLF7 downregulation improves EndMT in high glucose-treated HUVECs	(128)
KLF9	GCs-associated diabetes mellitus	Dexamethasone-induced Krüppel-like factor 9 expression promotes hepatic gluconeogenesis and hyperglycemia	Cui A et al.	2019	Dexamethasone induces KLF9, PGC-1α, Pck1, and glucose production related genes sequentially	Increases gluconeogenesis and blood glucose	(120)
	GDM	Interference of KLF9 relieved the development of gestational diabetes mellitus by upregulating DDAH2	Chen WX et al.	2021	Interference of KLF9 could hinder the development of GDM by alleviating oxidative stress, inflammatory responses, and apoptosis through upregulating DDAH2	KLF9 could regulate DDAH2 expression negatively by binding to the DDAH2 promoter	(121)
KLF11	NDM and MODY	Disruption of a novel Kruppel-like transcription factor p300-regulated pathway for insulin biosynthesis revealed by studies of the c-331 INS mutation found in neonatal diabetes mellitus	Bonnefond A et al.	2011	Induces the c-331KLF site through the p300-mediated pathway, and the transcription of INS	KLF11 is the activator of this site, mutations in this site can cause disease	(129)
	T2DM	Involvement of KLF11 in Hepatic Glucose Metabolism in Mice via Suppressing of PEPCK-C Expression	Zhang H et al.	2014	KLF11 inhibits hepatic glucose production and lowers blood glucose by decreasing PEPCK-C expression in mRNA and protein	KLF11 is a vital physiological regulator of hepatic gluconeogenesis	(130)
	Type 1B diabetes	KLF11 variant in a family clinically diagnosed with early childhood-onset type 1B diabetes	Ushijima K et al.	2019	His418Gln-KLF11 competes with WT-KLF11 for binding to cofactors, which may be related to the retention of activity for binding to cofactors	Specific variants of KLF11 with dominant-negative effects underlie incomplete penetrance in early childhood-onset type 1B diabetes	(131)
KLF14	T2DM	Association of KCNQ1 and KLF14 polymorphisms and risk of type 2 diabetes mellitus: A global meta-analysis	Wang J et al.	2014	Regulates important genes related to insulin resistance	Associated with T2DM susceptibility	(122)
		The association of type 2 diabetes loci identifed in genome qide association studies with metabolic syndrome and its components in a Chinese population with type 2 diabetes	Kong X et al.	2015	Regulates important genes associated with insulin resistance	Associated with T2DM susceptibility	(123)
KLF15	T2DM	Role of KLF15 in regulation of hepatic gluconeogenesis and metformin action	Takashima et al.	2010	Metformin efficiently downregulates the abundance of KLF15 in cells by both suppression of its mRNA and degradation of its protein	Involved in metformin induced gluconeogenesis inhibition	(124)
		Genetic Variation in Kruppel like Factor 15 Is Associated with Left Ventricular Hypertrophy in Patients with Type 2 Diabetes: Discovery and Replication Cohorts	Patel SK et al.	2017	Genetic variation may disrupt the potential of KLF15 to suppress hypertrophic transcription factors	KLF15 SNP rs9838915 A allele as the marker of left ventricular hypertrophy in patients with T2DM	(89)
		KLF15 negatively regulates cardiac fibrosis by which SDF-1β attenuates cardiac ffbrosis in type 2 diabetic mice	Tian YY et al.	2021	SDF-1β inhibits cardiomyocyte fibrosis through its receptor CXCR7-mediated activation of the p38β MAPK signaling pathway	KLF15 negatively regulates cardiac fibrosis through SDF-1β in T2DM	(71)

T2DM, type 2 diabetes mellitus; GDM, gestational diabetes mellitus; EndMT, endothelial-to-mesenchymal transition; HUVECs, human umbilical vein endothelial cells; NDM, neonatal diabetes mellitus; MODY, maturity onset diabetes in young.

**Figure 4:**
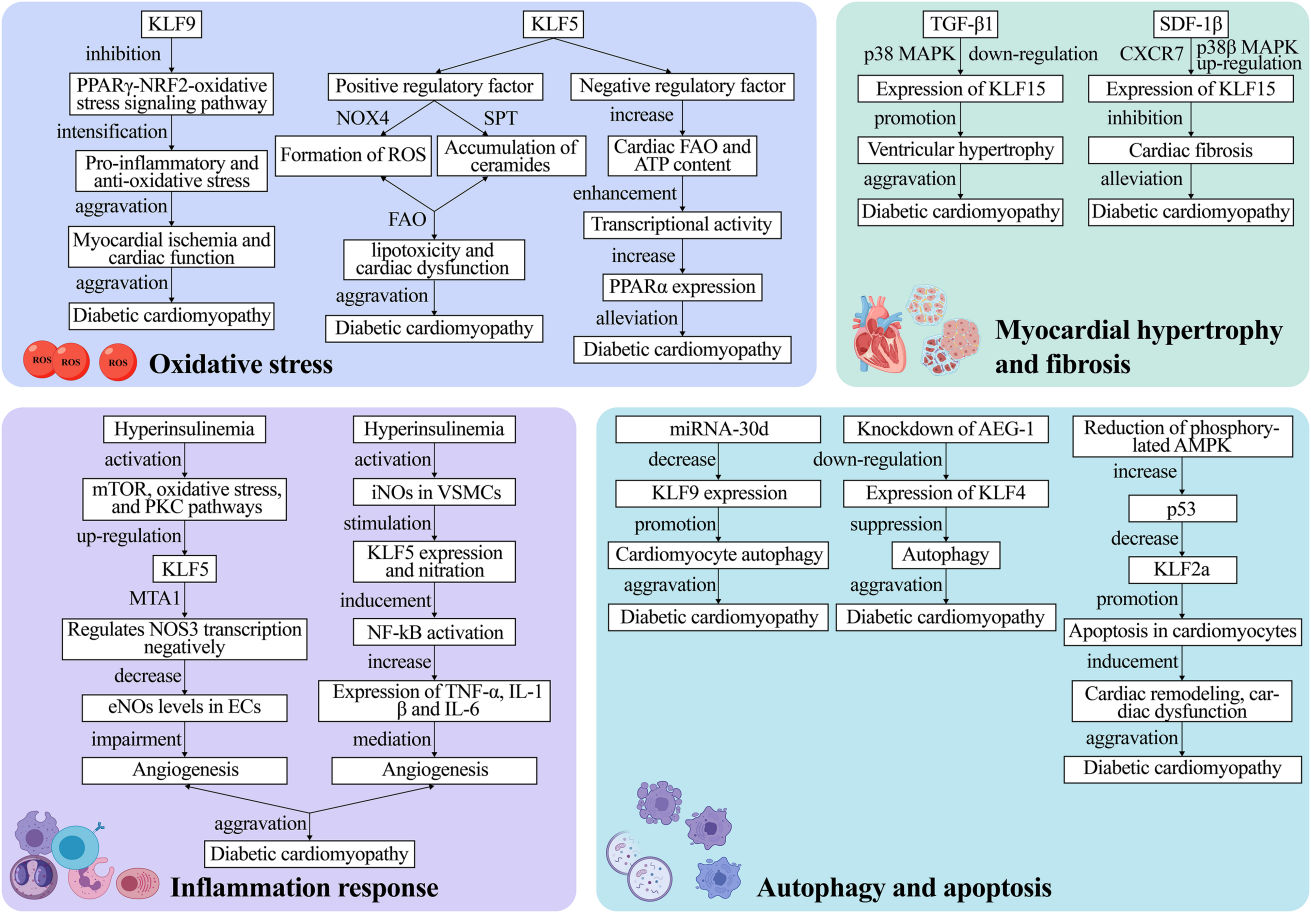
The mechanism of KLFs in regulation of diabetic cardiomyopathy. The pathophysiological process of diabetic cardiomyopathy mainly comprises four major pathways: oxidative stress, inflammation, myocardial hypertrophy and fibrosis as well as cell autophagy and apoptosis. These processes lead to irreversible heart failure. KLFs, as upstream and downstream regulators, can exacerbate or alleviate diabetic cardiomyopathy by targeting various metabolic pathways or signaling pathways, and the specific mechanisms of their action are presented in the figure. NOX4, NADPH-oxidase 4; ROS, reactive oxygen species; FAO, fatty acid oxidation; PPARα, peroxisome proliferator-activated receptor; mTOR, mammalian target of rapamycin; PKC, protein kinase C; eNOS, endothelial nitric oxide synthase; ECs, endothelial cells; iNOS, inducible nitric oxide synthase; VSMCs, vascular smooth muscle cells; p38 MAPK, p38 mitogen-activated protein kinase; SDF-1β, stromal cell-derived factor-1β; TGF-β1, transforming growth factor-β1; AEG-1, astrocyte elevated gene-1.

While genetic susceptibility plays a significant role in the pathogenesis of T2DM, the identified genes influencing susceptibility to T2DM are still limited (133, 134). Kanazawa et al. (127) found that KLFs not only play vital roles in cellular differentiation and tissue development but are also implicated in the pathogenesis of T2DM. In their study, they genotyped 33 single nucleotide polymorphisms (SNPs) in 12 KLF genes in T2DM patients and found a direct association between an allele of the SNP site in the second intron of KLF7 and T2DM, suggesting KLF7 as a novel candidate gene for genetic susceptibility to T2DM and its role in promoting the development of diabetic cardiomyopathy. Diabetes mellitus is an independent predictor of left ventricular hypertrophy (LVH), but not all diabetic patients develop LVH, indicating genetic components are involved. A clinical study investigated the association between the KLF15 gene and LVH in T2DM patients. Patel et al. (89) prospectively recruited 318 T2DM patients without known cardiac disease for transthoracic echocardiographic evaluation and genotyping for two KLF15 SNPs (rs9838915 and rs6796325). They found that the A allele of the rs9838915 SNP in the KLF15 gene was associated with increased left ventricular mass in patients, providing more accurate risk stratification for developing HF. Diabetic cardiomyopathy pathophysiology is multifaceted and involves complex metabolic pathways, with the main pathological features being myocardial hypertrophy and fibrosis, inflammation, cellular autophagy and apoptosis, and elevated OS markers (135–137). Myocardial hypertrophy makes the myocardium less compliant, causing diastolic dysfunction, which ultimately results in arrhythmia, HF, and even SCD (138, 139). In these processes, KLFs regulates the upstream and downstream pathways (Figure 4).

#### 4.2.1 Oxidative stress

Multiple studies have discovered that OS mediated by excessive Reactive Oxygen Species (ROS) levels is the pathogenic mechanism underlying type 1 and type 2 diabetes-related cardiomyopathy (140–142). The PPARγ-NRF2-OS signaling pathway stimulates cell survival signals, regulates autophagy, and exerts cardio-protective effects in cardiomyocytes (143). On the other hand, KLF9 aggravates ischemic injury in cardiomyocytes by exerting pro-inflammatory and anti-oxidative stress effects and deteriorates cardiac function by inhibiting PPARγ expression and transcriptionally lowering NRF2 expression and nuclear translocation (68, 69).

Diabetic cardiomyopathy is also associated with altered Fatty Acid Oxidation (FAO). The cardiomyocyte KLF5 can regulate cardiac FAO and induce *de novo* synthesis of ceramides through various pathways such as NADPH-Oxidase 4 (NOX4)-mediated ROS formation or SPT-mediated ceramide accumulation, leading to lipotoxicity and cardiac dysfunction (11). Correction of hyperglycemia with the SGLT2 inhibitor dapagliflozin reverses KLF5 expression in early diabetes (63). While KLF5 appears detrimental to cardiac function, another study proposed that transient KLF5 over-expression could be beneficial (63). Specifically, they discovered that cardiomyocyte-specific KLF5-deficient aMHC-KLF5-/- mice progressively developed cardiac dysfunction with signs of DCM. This outcome may be linked to the inhibition of cardiac Peroxisome Proliferator-Activated Receptor-α (PPARα) expression and reduced KLF5 transcriptional activity (64, 65). In the heart, KLF5 positively regulates FAO through PPAR transcription (66). Furthermore, KLF5 transcriptionally activates PPARα and decreases with PPARα gene expression (67). Additionally, cardiomyocyte-specific KLF5 ablation decreases cardiac FAO and ATP content, as well as transcriptional activity, ultimately triggering cardiac dysfunction. The above-mentioned findings imply that KLF5 has a dual function that could be exploited to treat or even reverse cardiomyopathy. However, this hypothesis requires further verification in future clinical trials.

#### 4.2.2 Inflammation response

Hyperinsulinemia and hyperglycemia could negatively affect angiogenesis by impairing the Endothelial Cell (EC) and Vascular Smooth Muscle Cell (VSMC) proliferation and transport. It has been reported that KLF5 induces vascular inflammation in diabetic VSMCs (144). Specifically, this process is divided into two parts: Negative regulation of endothelial Nitric Oxide Synthase (eNOS) expression and activation of inducible Nitric Oxide Synthase (iNOS) production. Inhibiting insulin signaling activates the mammalian Target of Rapamycin (mTOR), Protein Kinase C (PKC) pathways, and OS, up-regulates KLF5 along with MTA1, and negatively regulates NOS3 transcription, thereby leading to reduced eNOS levels in ECs, which impairs *in vitro* and *in vivo* angiogenesis (144). On the other hand, hyperglycemia activates iNOS in VSMCs, which, in turn, stimulates KLF5 expression and nitration. Stimulated KLF5 binds to NF-κB, inducing NF-κB activation, which increases TNFα, IL1β, and IL-6 expression and mediates vascular inflammation (145).

#### 4.2.3 Myocardial hypertrophy and fibrosis

Myocardial hypertrophy and cardiac fibrosis are prominent pathological remodeling features in hemodynamic or neurohormonal stress induced diabetic cardiomyopathy. Myocardial hypertrophy causes cardiac systolic and diastolic dysfunction, and KLF15 is a crucial negative regulator of diabetic cardiomyopathy-induced cardiac dysfunction and myocardial fibrosis (24, 72). Leenders et al. (73) discovered that TGF-β1 downregulates KLF15 in a p38 Mitogen-Activated Protein Kinase (p38 MAPK)-dependent manner and promotes ventricular hypertrophy. It was reported that KLF15 negatively regulated cardiac fibrosis in mice with type 2 diabetes, and the cardio-protective effects of Stromal Cell-Derived Factor-1β (SDF-1β) are mediated by binding to the CXCR7 receptor and the p38β MAPK-mediated upregulation of KLF15 (71). Furthermore, Takashima et al. (124) demonstrated that KLF15 plays an emerging role in regulating energy production and amino acid degradation. Gu et al. (146) recently found that the amide alkaloid Piperlongumine (PLG) extracted from piper longum exerted anti-hypertrophic and anti-fibrotic effects after angiotensin II (Ang II) treatment. This mechanism entails reduced levels of phosphorylated Akt and transcriptional regulation of fibrotic gene expression. Overall, this study enhances our understanding of the use of natural compounds for the targeted treatment of clinical diseases, implying that the co-mediation of the negative regulator KLF15 with natural compounds may be an entirely new therapeutic strategy for cardiomyopathy treatment in the future.

#### 4.2.4 Autophagy and apoptosis

Autophagy, a dynamic intracellular degradation mechanism, is a highly conserved eukaryotic cellular process that plays an important role in maintaining intracellular homeostasis as well as the synthesis, degradation, and recycling of cellular products. Growing evidence suggests that autophagy exerts a beneficial effect on diabetic hearts (147–150). Zhang and Wang recently found that decreased autophagic activity in cardiomyocytes is closely associated with diabetes-related cardiomyopathy and that restoring autophagy levels hinders diabetic cardiomyopathy development (70). According to research, KLF9 is a target gene of miR-30d that correlates negatively with miR-30d expression. Therefore, reduced KLF9 expression regulates autophagy in cardiomyocytes and exacerbates diabetic cardiomyopathy. Furthermore, the SGLT-2 inhibitor promoted cardiomyocyte autophagy by blocking miRNA-30d expression, and miRNA-30d negatively regulated KLF9, thereby improving cardiac function in diabetic cardiomyopathy mice (70). Additionally, KLF4 upregulation exacerbates cardiomyocyte autophagy; hence, aggravating diabetic cardiomyopathy. For the first time, Zhao et al. (62) found that the Astrocyte Elevated Gene-1 (AEG-1) can modulate autophagy in diabetic cardiomyopathy by regulating KLF4 expression, which is expected to be a new therapeutic target for diabetic cardiomyopathy treatment.

Increased cardiomyocyte apoptosis was found to be a major cause of systolic and diastolic dysfunction in diabetic models (151). Therefore, inhibiting cardiomyocyte apoptosis may effectively prevent myocardial remodeling in diabetic cardiomyopathy (152–154). It has been reported that KLF2a exerts cardio-protective effects through the AMPK-p53-KLF2a pathway. In a Streptozocin (STZ)-induced hyperglycemic zebrafish model, Wang et al. (61) found that decreasing phosphorylated AMPK elevated p53, leading to KLF2a downregulation, which in turn, promoted cardiomyocyte apoptosis and induced cardiac remodeling and dysfunction. KLF10 participates in the regulation of various aspects of tissue homeostasis and cellular functions, including proliferation, differentiation, and apoptosis (155, 156). In a study by Kong et al. (157), loss-of-function analyses of the zebrafish homologs of human KLF10 were conducted using antisense morpholino (MO). The results revealed that embryos injected with KLF11b-MO exhibited developmental retardation and cell death, whereas those injected with KLF11a-MO did not display significant abnormalities in development. Moreover, embryos co-injected with KLF11b-MO and p53-MO showed reduced apoptosis. These findings suggest that KLF10 serves as a critical negative regulator of p53-dependent transcription, and the KLF10/p53 complex contributes to apoptosis and hence the maintenance of tissue homeostasis.

### 4.3 Dilated cardiomyopathy

Dilated Cardiomyopathy (DCM), a multifactorial disorder associated with genetics, immunity, infection, and the environment, is primarily characterized by heart enlargement and systolic dysfunction (51, 158, 159). The prevalence of DCM is at 1:2,500 in 2013 (160) and the diseases is always progressive, eventually leading to irreversible HF and sudden death. The annual SCD incidence in DCM is 2%–4% (161, 162). Given that the disease is multifactorial, effectively targeting treatment to its etiology has become a major research focus (158, 163, 164). In this regard, multiple studies recently demonstrated the critical functions of KLFs in DCM, including regulating molecular mechanisms contributing to DCM progression.

Multiple mechanisms such as inflammation, Endoplasmic Reticulum Stress (ERS), and mitochondrial dysfunction play vital roles in the DCM-associated ventricular dysfunction progression and HF development. Specifically, it has been reported that myocardial injury triggers an inflammatory response. Zhang et al. (74) found that the KLF2 overexpression-derived Extracellular Vesicles (EVs) reduced cardiac inflammation and improved left ventricular dysfunction in DCM mice by inhibiting Ly6C^high^ monocyte mobilization via targeting the CCR2 protein. High KLF2 expression in ECs exerts an anti-inflammatory effect, implying that KLF2 may be a potential therapeutic target for DCM treatment.

Furthermore, growing evidence highlights the crucial role of genetic defects in the pathogenic mechanism of DCM (165). As one of the mutation carriers of disease-causing genes, KLF5 mutations co-segregating with DCM have shown complete penetrance in this family, and genetically compromised KLF5 is highly susceptible to DCM (76). Notably, KLF13 is also co-segregated from DCM. Also known as FKLF2/BTEB3/RFLAT-1/RFLAT1/NSLP1, KLF13 is a new DCM susceptibility gene localized on chromosome 15q13.3. Guo et al. (77) conducted sequencing analysis focusing on specific genes within the localization region of human chromosome 15q13.1-q13.3 and identified three mutations that co-segregated with the dilated cardiomyopathy (DCM) phenotype with complete penetrance. These mutations, namely c.430G>T (p.E144X), c.580G>T (p.E194X), and c.595T>C (p.C199R), exhibited impaired transactivation of the target genes ACTC1 and MYH7, both individually and in combination with GATA4, a gene known to induce DCM. Furthermore, these mutations displayed reduced binding capacity to the promoters of ACTC1 and MYH7. Furthermore, the intracellular distribution of E144X mutant KLF13 was disrupted. In summary, detecting the aforementioned new susceptibility genes and loci offers new insights into the DCM molecular pathogenesis and may provide guidance for precision medicine for DCM.

### 4.4 Desmin-related cardiomyopathy

Desmin, a major intermediate filament protein in cardiomyocytes, is a fundamental component of the cellular structure and the Purkinje fibers of the conduction system (166, 167). Desmin is encoded by the DES gene (OMIM *125660), and mutations in this gene are hypothesized to disrupt mechanical stress in the muscle, leading to rhabdomyolysis (168–170) Additionally, desmin is crucial in balancing ROS generation with antioxidant defense. According to research, MnSOD overexpression and/or catalase are the major antioxidant defense systems that can reduce ROS production and improve cardiac function in desmin-deficient mice hearts (171).

Desmin-Related Cardiomyopathy (DRCM) presents early with lower extremity muscle weakness and gait disturbances before progressing to the proximal, respiratory, fascial, and cardiac muscles, eventually leading to arrhythmias and congestive heart failure. Notably, heart defects occasionally precede skeletal muscle defects (167, 172, 173). A meta-analysis of 159 patients with 40 different DES gene mutations revealed that more than 70% of carriers exhibited myopathy or muscular weakness and that about 50% of carriers had cardiomyopathies. In another study, up to 60% of patients had a cardiac conduction disorder or arrhythmias, such as an atrioventricular block (174). Except for symptomatic treatments for alleviating the symptoms, especially in the cardiac area focusing on cardiovascular complications (175, 176), there are currently no targeted therapies for DRCM. Khudiakov et al. (79) delivered four reprogramming factors (OCT4, KLF4, SOX2, and CMYC) with Sendai virus from patient-specific fatty tissue-derived pluripotent Mesenchymal Stromal Cells (MSCs) carrying heterozygous splice site mutations in the DES gene using a non-integrative reprogramming approach, ultimately yielded a human iPSC line. Establishing an iPSC line holds promise for addressing DRCM and for the future development of innovative drugs. Several KLFs may share similar regulatory mechanisms across various muscle tissues. Moreover, within the same muscle tissue, there could be synergistic or antagonistic interactions among KLFs, crucial for muscle tissue development and functional regulation. For instance, KLF2, KLF4, KLF10, and KLF15 have been implicated in these processes. In mouse skeletal muscle cells, the extracellular signal-regulated kinase 5 (ERK5) signaling pathway can induce the upregulation of KLF2 and KLF4 genes via the Sp1 transcription factor. This activation subsequently promotes the expression of nephronectin (Npnt) genes, facilitating skeletal muscle cell fusion through enhanced cell-matrix adhesion (78). KLF15, on the other hand, can stimulate the expression of the slow-twitch fiber gene Myh7 (MHC-β/slow) by targeting the nuclear factor of activated T-cells, cytoplasmic 1 (NFATc1) gene, thereby positively regulating skeletal muscle differentiation (82). Conversely, KLF10 inhibits myoblast proliferation by suppressing the function of pro-proliferative signaling molecules (80) and the expression of cyclin (81). Most mutations associated with DRCM, such as those affecting CRYAB and BAG3, are missense or small deletion mutations. The formation of aggregates appears to be a significant trigger for DRCM. However, the downstream effects of these mutations are diverse and heterogeneous (177). While a direct link between DRCM and KLFs has yet to be established, ongoing advancements in molecular and cellular biology hold promise for the development of molecular therapies for DRCM. Targeting the regulation of DES gene expression by KLFs may emerge as a promising strategy for reducing the expression of mutant DES alleles and mitigating DRCM pathology.

### 4.5 Hypertrophic cardiomyopathy

Hypertrophic Cardiomyopathy (HCM), a relatively common monogenic heart disease with autosomal dominant inheritance, is characterized by ventricular hypertrophy on echocardiography, mostly in the left ventricle (51, 178, 179). The disease was once assumed to be a sudden exercise death disease that primarily affected young men, but is no longer restricted to young males. According to the 2018 global hypertrophic cardiomyopathy burden statistics, HCM affected 6.3 billion people or 88% of the world's population (178). Auspiciously, HCM treatment has progressed from limited palliative pharmacotherapy and occasional high-risk surgery to more definitive screening tools and highly efficient interventional therapies, resulting in a 0.5% annual reduction in morbidity and mortality (180–183).

Although HCM is usually a non-progressive disease, HF symptoms may occur or worsen at any age, most often in middle-aged individuals (184, 185). Efficient management of myocardial hypertrophy is crucial given its potential progression to HF. One significant hallmark of this transition is the shift in myocardial substrate preference from FAO to glycolysis, resembling an embryonic metabolic profile. KLFs have emerged as key transcriptional regulators in pathological cardiac hypertrophy and metabolism. For instance, Wang et al. (86) demonstrated that both cardiac-specific knockdown and overexpression of KLF7 disrupt the balance between glycolysis and fatty acid metabolism, leading to cardiac hypertrophy and myocardial fibrosis. Additionally, KLF4 serves as a central transcriptional regulator of cardiac metabolic function and mitochondrial dynamics, reactivating fetal cardiac genes during hypertrophic development (83, 84). *In vivo* studies have shown that KLF4 can modulate isoproterenol-induced cardiac hypertrophy by regulating myocardin (MYOCD) expression and activity, and cardiomyocyte-specific knockdown of KLF4 exacerbates hypertrophy (85). Oxytocin (OT), a hormone involved in cardiovascular homeostasis, mitigates cardiac hypertrophy by targeting the lncRNA GAS5/miR-375-3p/KLF4 axis to inhibit the PI3K/AKT pathway (186). Moreover, TIEG1 has been identified as a critical player in cardiac hypertrophy (87). The TGFb Inducible Early Gene-1 (TIEG1) is classified as a member of the KLF10 family and is involved in gene transcription regulation (42). Subramaniam et al. (88) studied male mice aged 4–16 months and found that TIEG1-/- mice exhibited cardiac hypertrophy compared to wild-type animals. This finding demonstrates that TIEG1, a gene transcription inducer or repressor, can inhibit cell proliferation and induce apoptosis by activating the TGF-β/Smad pathway. KLF15 is a pivotal regulator of cardiac metabolic homeostasis, primarily governing myocardial lipid flux. It plays a significant role in controlling the transcriptional network associated with cardiac metabolism (187). Notably, KLF15 exerts its regulatory effects by negatively modulating pro-hypertrophic and pro-fibrotic markers such as brain natriuretic peptide (BNP) and connective tissue growth factor (CTGF) through the p38-MAPK signaling pathway. This pathway has been identified as a crucial negative regulator of cardiac hypertrophy (90). Deletion or inhibition of KLF15 reduces the suppression of pro-hypertrophic factors and the stimulation of fibrotic signaling pathways, resulting in Left Ventricular Hypertrophy (LVH) development (58, 89).

### 4.6 Ischemic cardiomyopathy

Ischemic Cardiomyopathy (ICM), generally referred to as systolic left ventricular dysfunction in the context of obstructive CAD, is the leading cause of HF worldwide (188, 189). Ventricular dysfunction in CAD patients is usually caused by the irreversible loss of viable myocardium after Acute Myocardial Infarction (AMI) (189, 190). According to research, KLF5 regulates ceramide accumulation and lipid metabolism after Myocardial Infarction (MI) (91). Furthermore, KLF5 is involved in the aggravation of Ischemic Heart Failure (IHF), and induced KLF5 increases the expression of some regulatory factors [including Serine-Palmitoyl-Transferase-Long-Chain-Base-Subunit (SPTLC)1 and SPTLC2], and ceramide biosynthesis, leading to systolic dysfunction and eccentric remodeling, thereby decreasing ejection fraction and exacerbating IHF (91).

Following infarction, the remaining surviving segmental myocardium adapts to chronic perfusion insufficiency by reducing its energy requirements blocking myofibrillar protein expression, with the heart preferentially relying on glucose absorption rather than fatty acids for energy supply. This gene expression reprogramming via fluctuations in the cellular microenvironment is known as epigenetic reprogramming (191–193). It has been reported that KLF15 is an upstream regulator of metabolic gene expression, and that it is negatively regulated by EZH2 (an epigenetic regulator) in a SET structural domain-dependent manner for cardiac metabolism and structural reprogramming (92). Additionally, KLF15 suppresses gene expression and negatively regulates adverse cardiac remodeling and hypertrophy in ICM progression (72, 92, 96). Specifically, KLF15 antagonizes the expression of various genes actively involved in cardiac remodeling. Rogers and Otis studied the mechanism of resveratrol (an antioxidant) in ischemic heart treatment. They found that chronic myocardial ischemia significantly reduced KLF15 expression, which then increased the transcription of TGF-β1 and Nox4 mRNA, and that sustained TGF-β1/Nox4 signaling led to the production of large amounts of oxidants, which aggravated OS in diseased hearts, as well as myocardial fibrosis and hypertrophy (194). Haldar et al. (93) reported that the KLF15-p53-p300 pathway may also be a therapeutic target in CVD treatment. Specifically, they found that KLF15 activation inhibits the p300-mediated p53 acetylation. On the other hand, KLF15 deficiency causes p53 hyperacetylation in the aorta and heart (93–95). Mice lacking KLF15 develop aortopathy and cardiomyopathy in p53-dependent and p300 acetyltransferase-dependent manners.

### 4.7 Obesity-associated cardiomyopathy

Globally, the morbidity and mortality levels due to HF have been on the rise, making HF a major threat to human health. The etiology of HF is multifactorial, and studies have shown that uncorrected overweight and obesity may be independent risk factors for development of CVDs (195). Evidence from previous experimental, clinical, and epidemiological studies has pointed to the existence of a distinct disease entity known as “obesity cardiomyopathy”, which occurs independently of other CVD risk factors such as hyperlipidemia, hypertension, and diabetes mellitus (196). “Obesity cardiomyopathy” refers to metabolic and functional abnormalities of the heart caused by obesity alone (197, 198). In general, chronic obesity is strongly associated with myocardial remodeling, and its clinical features may range from LVH and myocardial fibrosis which eventually evolve to HF (199–201). Obesity also affects myocardial electrophysiology, which increases the prevalence of atrial fibrillation (202, 203).

Multiple studies have shown that berberine prevents heart disease, controls cardiac remodeling (204–207), and ameliorates KLF4-dependent obesity-related cardiac damage (97, 98). Ding et al. (99) treated high fat diet-induced obese mice with berberine and found that this drug ameliorated myocardial mitochondrial biogenesis and activity. However, KLF4 silencing reduced the mitochondrial mass, ATP production, oxygen consumption, and lipid metabolism, which were upregulated by berberine treatment. It also reversed berberine's structural benefits on cardiac anti-hypertrophic and fibrotic, anti-inflammatory, and antioxidant properties. In summary, these findings suggest that KLF4 indirectly mediates obesity-associated cardiac injury, controls myocardial remodeling, and confers protection on the heart.

## 5 Summary and outlook

Cardiovascular diseases (CVDs), including cardiomyopathy, contribute to high morbidity and mortality worldwide. This calls for investigations to identify targeted strategies for preventing and treating patients with cardiomyopathy. Unfortunately, the etiology of cardiomyopathies has not been fully clarified given its multifactorial nature. Furthermore, the pathophysiological mechanisms underlying cardiac dysfunction and myocardial remodeling in various types of cardiomyopathies are highly intricate and necessitate additional research. As such, the exploration of novel targets or regulators, both upstream and downstream, that exert direct or indirect influence on the progression of cardiomyopathy has become of paramount importance. In recent years, there has been renewed research interest on the role of Krüppel-Like Factors (KLFs) in various biological processes and conditions such as myocardial diseases, such as oxidative stress, inflammatory responses, myocardial hypertrophy and fibrosis and apoptosis. KLFs are a group of DNA-binding proteins associated that can activate or suppress genes, stimulate cell growth, differentiation, apoptosis, and biological processes related to tissue development and maintenance. Here, we review the mechanisms underlying the development of 7 cardiomyopathies focusing on the involvement of 18 members of the KLFs family in terms of their classification. For example, KLF13, localized on chromosome 15q13.3, is a novel gene that increases the risk of cardiomyopathy (DCM), whereas KLF15 can prevent diabetic cardiomyopathy-induced cardiac dysfunction and myocardial fibrosis as well as hypertrophic cardiomyopathy (HCM) by repressing pro-hypertrophic transcription factors to attenuate LVH.

While numerous studies, including those conducted in animal models, cell trials, and clinical settings, have highlighted the importance of KLFs as crucial regulators or markers of cardiomyopathies, the precise pathophysiological roles of certain KLF members in different types of cardiomyopathy remain poorly understood. For instance, it is unclear whether KLF2 and KLF4 participate in gestational diabetes mellitus-associated cardiomyopathy. Therefore, there is a need for more comprehensive investigations to elucidate the signaling pathways modulated by KLFs in various types of cardiomyopathies. These pathways may include, but are not limited to, the Hippo/Yap signaling pathway, Wnt signaling, Hedgehog (Hh) signaling, Notch pathway, and Mitogen-activated protein kinases (MAPKs) signaling. In the article, we describe the importance of KLFs in cardiomyopathies and hope to provide new ideas regarding the cellular and molecular biological mechanisms involved in the regulation of KLFs and reveal new insights into development of precision medicine for patients with cardiomyopathies. In future, we predict that pharmacology and invasive procedures will no longer be the only means of treating cardiomyopathy, preventing sudden death, or heart transplantation. Therefore, a better understanding of the molecular genetics of cardiomyopathy wil boost the development of potential therapeutic targets for cardiomyopathy.
